# Outcomes after appendectomy in children with acute appendicitis treated at a tertiary paediatric centre: results from a retrospective cohort study

**DOI:** 10.1007/s00423-020-01976-y

**Published:** 2020-08-28

**Authors:** Max Knaapen, Paul van Amstel, Tim van Amstel, Sarah-May M. L. The, Roel Bakx, Ernest L. W. E. van Heurn, Ramon R. Gorter

**Affiliations:** Department of Paediatric Surgery, Emma Children’s Hospital, Amsterdam UMC, University of Amsterdam & Vrije Universiteit Amsterdam, PO Box 22660, 1100DD Amsterdam, The Netherlands

**Keywords:** Appendicitis, Paediatric surgery, Appendectomy, Post-appendectomy complications

## Abstract

**Purpose:**

In general, an appendectomy is presumed to have a limited burden of disease. However, in current literature, reported complication rates vary. This study aims to provide additional insights in the incidence of post-appendectomy complications in children with acute appendicitis.

**Methods:**

This retrospective cohort study included children (0–17 years old) that underwent appendectomy at our tertiary referral centre for suspected acute appendicitis (January 2011–December 2018). Children referred to our centre, and those that underwent non-operative treatment were excluded. Post-appendectomy complications were recorded from electronic medical charts using predefined definitions and classified as severe (Clavien-Dindo III–IV) or less severe (Clavien-Dindo I–II).

**Results:**

A total of 131 children were included. Simple and complex appendicitis was diagnosed in 66 (50%) and 60 (46%) children, respectively. A non-inflamed appendix was seen in five (4%) children. One or more complications were identified in 33 (25%) patients. Eight (12%) children with simple appendicitis developed a complication, three of these were severe. In children with complex appendicitis, 23 (38%) children developed a complication, 14 of these were severe.

**Conclusion:**

This study shows a high rate of complications compared with current literature, both in children with simple and complex appendicitis. This is probably the result of our definition of complications and being a tertiary referral centre receiving more severe appendicitis cases. However, these results still show that appendectomy is not always a routine procedure with only few complications. Substantiating the need to keep optimizing treatment for children with appendicitis.

## Introduction

Acute appendicitis is a common gastrointestinal disease in children. Roughly one in every 13 people will be afflicted by appendicitis at some point in their life, and in around one-third of all people, this will be during childhood or adolescence [1]. In recent years, the classification of acute appendicitis into two types has become well established: simple appendicitis (or non-perforating/uncomplicated) and complex appendicitis (or necrotizing/perforating) [2]. The incidence of these two types varies significantly across ages. In the paediatric population, simple appendicitis accounts for the majority of cases, approximately in 65% [3, 4]. For over one hundred years, the appendectomy has been the standard of care for all types of acute appendicitis; however, time has not stood still. The open appendectomy has mostly been replaced by the laparoscopic appendectomy, complicated appendicitis is now routinely treated with a postoperative course of antibiotics [5], and even the dogma that suspected appendicitis should be operated on as quickly as possible to prevent perforation is being replaced by the option to delay surgery in selected cases for up to 24 h [6]. In the search for the optimal treatment for appendicitis, we need information on current post-appendectomy complication rates. However, available evidence shows a large variance and is of varying quality. The reported complication rates range from 5 up to 15% of all paediatric cases, and even 29% in the subgroup of children with complex appendicitis [4, 7–9]. In recent years, the interest for non-operative treatment of simple appendicitis, with antibiotics, as an alternative for appendectomy is increasing. Currently we are awaiting the results of several ongoing randomized controlled trials in children [10–13], but in the meantime we can benefit from more information on the risks of appendectomy as current practice. Therefore, the purpose of this study is to provide additional insights in the incidence of post-appendectomy complications in order to substantiate the need to improve treatment for children with appendicitis.

## Materials and methods

In this retrospective cohort study, we included children (0–17 years old) that underwent appendectomy at our paediatric surgical centre for suspected acute appendicitis between January 2011 and December 2018. Eligible patients were identified using ICD codes for acute appendicitis, acute abdomen, intra-abdominal abscess, and general abdominal pain. Patients that were non-operatively treated for suspected acute appendicitis, and those with a finding of a non-inflamed appendix during laparoscopy (and thus did not undergo appendectomy) were excluded. We are a tertiary referral centre; however, we also have a regional function for all children presenting at our centre. In this study only children that primarily presented at our centre were included, any referrals were excluded to minimize the inclusion of patients with more co-morbidities or more severe forms of appendicitis. Children referred for treatment of post-appendectomy complications after they underwent appendectomy elsewhere were also excluded.

### Intervention

In our centre, both the laparoscopic and open approach for appendectomy are used, although the use of open appendectomy has become rare over the years. In all cases, antibiotic prophylaxis consisting of a single dose of cefazolin (30 mg/kg) was administered 30 min prior to incision. Open appendectomy was performed through a McBurney’s incision and ligation of the appendiceal stump. For laparoscopic appendectomy the conventional three-port method was used. The use of endoloops or endostapler for appendiceal stump closure, suction, peritoneal washout or abdominal drains were all at the discretion of the surgeon. No standardized peritoneal cultures are taken. If intraoperatively there were signs of gangrene, perforation, purulent fluid, periappendicular contained phlegmon or an intra-abdominal abscess (IAA) broad-spectrum antibiotics (either the combination of amoxicillin/clavulanic acid (100/10 mg/kg/day) together with gentamicin (7 mg/kg/day) or cefuroxime (100 mg/kg/day) together with metronidazole (30 mg/kg/day)) were administered intravenously for 3–5 days. If none of the before mentioned was observed the child was discharged the next day without any postoperative antibiotics.

### Outcomes

Based upon the perioperative diagnosis and histopathological examination, patients were divided into three groups according to the classification by Bhangu et al. [2]; (1) Normal non-inflamed appendix, including acute intraluminal inflammation, (2) simple, non-perforated appendicitis, or (3) complex appendicitis, either gangrenous, perforated or with and intra-abdominal abscess.

The main outcome of this study is the number of patients with at least one post-appendectomy complication. The complications were scored using pre-defined definitions. When complications were encountered not fitting any of the predefined definitions they were scored after a group discussion. The definitions of the most common complications were:

- IAA: collection of fluid in the abdomen, confirmed by radiological imaging [14].
- Surgical site infection (SSI): Postoperative infection in the surgical area, classified as superficial or deep SSI’s [15]:
  - Superficial: Inflammation of the skin and subcutaneous tissue
  - Deep: Inflammation of deep soft tissues of the incision, such as the muscles, fascia or surrounding tissues
- Ileus : No resumption of diet within five days of intervention [4, 16].

Post-operative pain on itself was not considered a complication. The severity of the complications were classified according to the Clavien-Dindo scale [17, 18]. Complications were categorized in severe (Clavien-Dindo III–IV) or less severe (Clavien-Dindo I–II). Secondary outcomes were the length of hospital stay (in days) and the number of readmissions due to complications related to the appendectomy. Follow-up was performed by reviewing electronic patient records for readmission. No telephone follow-up was performed.

### Data extraction and analysis

Data was extracted from electronic patient records according to a standardized form using an electronic database, Castor Electronic Data Capture (Ciwit BV, 2016. Amsterdam, The Netherlands). One author (TvA) performed the data extraction and all patients were reviewed by another author (MK). In case of discussion patients were reviewed by a third author (RRG).

Descriptive statistics were performed using IBM SPSS version 25.0 (IBM Corp. Released 2017. Armonk, NY). For non-normally distributed continuous data we used medians with interquartile ranges (IQR). For normally distributed data means were reported.

## Results

### Population and general characteristics

During the study period, a total of 1411 patients were identified based on the ICD codes for acute appendicitis, acute abdomen, intra-abdominal abscess, and general abdominal pain. Of these, 1137 patients were excluded from our cohort because they were not treated for suspected acute appendicitis. Furthermore 72 patients with suspected acute appendicitis were excluded because they were referred to our hospital (mostly due to a very low age or to the severity of appendicitis) and 46 patients were excluded because they underwent appendectomy at another centre due to limited capacity at our tertiary centre. Additionally 17 patients were excluded because they underwent non-operative treatment, seven patients because a non-inflamed appendix was diagnosed during laparoscopy and no appendectomy was performed, and one patient because an appendiceal malignancy was found at histopathological examination. In total 131 patients were included with a mean age of 10 years (range 1–17 years) that primarily presented at our tertiary centre with a suspicion of acute appendicitis and subsequently underwent appendectomy. Appendectomy was performed laparoscopically in most patients, 118 of 131 (90%). Simple appendicitis was diagnosed in 66 of the 131 (50%) children and complex appendicitis in 60 of 131 (46%) children. At histopathological examination, a non-inflamed appendix was found in five of 131 (4%) children. General characteristics of the study cohort are shown in Table 1.

**Table 1:** Baseline characteristics all patients

	Total (*n* = 131)
Male (*n*, %)	74 (56%)
Age in years (mean, range)	10.4 (1–17)
Length in meters (median)	1.48
Weight in kg (median, range)	36 (12–100)
Appendicitis type	
Simple (*n*, %)	66 (50%)
Complex (*n*, %)	60 (46%)
Non-inflamed (*n*, %)	5 (4%)
Operative approach	
Open (*n*, %)	13 (10%)
Laparoscopic (*n*, %)	118 (90%)
Converted to open (*n*, %)	15 (13%)

*n* = number of patients

*kg* = kilogram

### Complications

Of the 131 included children, 33 (25%) developed one or more post-operative complications as displayed in Table 2. In children with simple appendicitis, 8 out of 66 (12%) children developed a complication, three of those were severe (Clavien-Dindo III–IV). Of the children with complicated appendicitis, 23 out of 60 (38%) children developed a complication, 16 of these were severe (Clavien-Dindo III–IV). In total we identified 17 different post-operative complications. Each of the complications required some form of treatment and, in most cases, an extended hospital stay. All complications for patients with simple and complex appendicitis are listed in Tables 3 and 4.

**Table 2:** Complication rate, length of stay and readmissions

	Simple (*n* = 66)	Complex (*n* = 60)
Complication present (*n*, %)†	8 (12%)	23 (38%)
Severe complications (Clavien-Dindo III–IV) (*n*, %)^a^	3 (5%)	14 (23%)
Less severe complications (Clavien-Dindo I–II) (*n*, %)^a^	5 (8%)	13 (22%)
Initial length of stay in days (median, IQR)	1.5 (1.0–2.0)	5.0 (4.0–6.5)
Readmissions (*n*, %)	4 (6%)	11 (18%)
Length of readmission in days (median, IQR)	3.5 (2.0–5.8)	4.0 (2.0–11.0)
Total length of stay in days (median, IQR)	1.5 (1.0–2.0)	5.5 (4.0–8.0)

*n* = number of patients

*IQR* = interquartile range

^†^Some patients suffered both a severe and less severe complication

**Table 3:** Complications in patients with simple appendicitis

	Simple (*n* = 66)
Severe complication (Clavien-Dindo III–IV)†	
1. Intra-abdominal abscess (*n*)	1
2. Incisional hernia (*n*)	1
3. ARDS (*n*)	1
Less severe complication (Clavien-Dindo I–II)†	
1. Wound infection (*n*)	2
2. Intra-abdominal abscess (*n*)	1
3. Urinary tract infection (*n*)	1
4. Abdominal pain, unknown origin (*n*)	1

*n* = number of cases

*ARDS* = acute respiratory distress syndrome

^†^Some patients suffered multiple complications

**Table 4:** Complications in patients with complex appendicitis

	Complex (*n* = 60)
Severe complication (Clavien-Dindo III–IV)†	
1. Intra-abdominal abscess (*n*)	9
2. Ileus (*n*)	4
3. Appendiceal stump leakage (*n*)	1
4. Incisional hernia (*n*)	1
5. Shock, unknown origin (*n*)	1
Less severe complication (Clavien-Dindo I–II)†	
1. Intra-abdominal abscess (*n*)	4
2. Antibiotic related complications (*n*)	3
3. Wound infection (*n*)	2
4. Postoperative infiltrate (*n*)	2
5. Fistula (*n*)	1
6. Allergic reaction thrombocyte transfusion (*n*)	1
7. Gastro-enteritis (*n*)	1
8. Atelectasis (*n*)	1
9. Candida diaper rash (*n*)	1
10. Urinary retention (*n*)	1

*n* = number of cases

^†^Some patients suffered multiple complications

Two out of the five patients with a non-inflamed appendix developed a complication. One developed an IAA which was initially percutaneously drained. However, after a few days, a laparotomy was necessary because the abscess would not subside. The second patient turned out to have a situs inversus with incomplete malrotation. Because of an ongoing ileus, a repeat laparotomy was necessary in which the entire colon was mobilized.

Readmission was necessary in 16 out of 131 (12%) patients. Reason for readmission was an IAA in twelve patients. One following an appendectomy with a non-inflamed appendix at histopathological examination, however, no alternative diagnosis could be found. Two patients were readmitted because of an incisional hernia (after 91 and 94 days) and one because of a urinary tract infection. The last patient was readmitted for an ileostomy reversal that was put in during the primary admission following an appendiceal stump leakage. As shown in Table 2, the median length of stay, including readmissions, for children with simple appendicitis was 1.5 days. For complex appendicitis, median length of stay was 5.0 days, and including readmissions 5.5 days.

## Discussion

In current literature, the reported overall incidence of post-appendectomy complications in children varies. Often an incidence of around 10% is reported [4, 7]; however, these numbers vary between five and 15% [8, 9, 19]. The overall complication rate of 25% in the study is high in comparison with the current literature. After evaluating the complication rate for both types of appendicitis (simple or complex), our complication rate was found to be high in both groups. The complication rate of 12% in children with simple appendicitis is substantial and high compared with other studies that report complication rates between 2 and 5% [4, 8, 9, 19, 20]. This can also be concluded for our subgroup of children with complex appendicitis, in which a complication rate of 38% was found, whereas current literature reports complication rates between 15 and 32% (depending on the operative approach) [4, 8]. As a tertiary paediatric centre, we treat a variety of complex gastrointestinal diseases and most complex cases are referred to our academic hospital. Moreover, many patients that are treated at our tertiary referral centre suffer from extensive comorbidity such as immune disorders and/or nephrological disorders requiring high doses of immunosuppressant medication. Therefore, our general population consists of complex patients with extensive comorbidity. We also receive appendicitis cases from other centres due to the age of the child, comorbidities or the severity of the appendicitis. We anticipated that inclusion of these children in our cohort could lead to selection bias resulting in a higher complication rate. Therefore, we excluded 72 children that were referred to us from other hospitals. The high complication rate in this study compared with the literature could also be explained by the definitions of postoperative complications used in this study. Whereas many other studies only report the most frequent complications (i.e. intra-abdominal abscess, wound infection, ileus), we reported any type of complication that needed a form of treatment. To assess the severity of complications, the Clavien-Dindo classification was used [17]. Even though this is the most frequently used classification for the severity of complications, its generalizability can be questioned. As an example, the threshold for ICU admittance varies between (academic and teaching) hospitals. Therefore, the development of a new (paediatric) complication severity classification could be of additional value for future studies. Universal reporting of the type of appendicitis and its complications, including universal definitions for these complications and their severity, is a key element to improve comparability of future studies. An important tool to promote in universal outcome reporting is the use of core outcome sets. These outcome sets are an agreed standardized set of outcomes that should be measured and reported in all trials investigating the optimal treatment of acute (simple) appendicitis. Apart from universal outcome reporting, well-defined and universally used definitions for these outcomes (such as complication definitions) are equally important [21].

Still the results of this study signify that, for a selected population, the appendectomy is not always without complications and there is still more work to be done to minimize post-appendectomy complications. This seems particularly relevant now that there is increasing interest in non-operative treatment with antibiotics of simple appendicitis as an alternative for appendectomy. However, in the adult population, varying results after non-operative treatment for acute simple appendicitis have been reported. Some studies show that non-operative treatment could potentially reduce the complication rate by 39–71%, whereas others report an event-free treatment success favourable to operative treatment [22–24]. Similar varying outcomes are reported in children, and a recent meta-analysis found an initial success rate of 90% without a significant difference in complications compared with appendectomy [25]; however, a different meta-analysis of the mostly the same studies concludes that NOT is less efficacious and associated with a higher readmission rate [26]. In this debate, universal reporting of complications is of crucial importance. Besides complications, non-operative treatment comes with a risk of recurrent appendicitis which in children lies between 10 and 20% [20]. Definitive answers will have to come from several randomized trials that are currently underway [10–13].

In our study, the risk of developing a post-appendectomy complication was triple for children with complex appendicitis compared with simple appendicitis. The debate on the optimal treatment for complicated appendicitis is also still ongoing. No consensus exists on how to treat children with an (large) appendicular infiltrate or abscess; many experts still prefer a direct appendectomy as it prevents a readmission for an interval appendectomy. However, some studies suggest that non-operative treatment of this particular patient group, consisting of clinically ill patients at presentation, is associated with significantly less complications [14, 27]. Considering the significant burden of disease that is associated with complex but also simple appendicitis, we need to keep looking for the optimal treatment for children with appendicitis.

### Strength and limitations

The results of this study should be interpreted with caution as our sample size is smaller than some of the other currently published studies. Also our study is retrospective and thus by nature prone to bias. This study has several strengths. Firstly, two independent researchers reviewed the complete electronic medical records of all included patients. This differs from many other (larger) studies that analyse discharge registrations or other routine databases [19, 28, 29]. Secondly, many studies only report the most common complications, for example only IAA’s, SSI’s, ileus and incisional hernias [4, 7, 30]. In this study, we chose to report any type of complication, but only if some form of treatment was warranted. By doing so, we attempted to limit any underreporting of complications. We cannot rule out that the high complication rate in our single centre study is the result of (inadequate) diagnostics or care in our centre. This could also be the result of a limited case load of only 131 patients in eight years that were operated by different paediatric surgeons. However, the actual case load at our centre is somewhat higher as we excluded a significant number of cases. Thorough review of the cases with complications in this cohort did not yield any directly preventable complications. Although the overall complication rate in our study was relatively high compared with other studies, some complications could have been missed, as we did not perform telephone follow-up at the time of data analysis. Routinely patients who experience any complication after treatment at our tertiary centre are referred back to us. However, it could be possible that they presented with complications at other hospitals.

As the risk of complications is much higher in children with complex appendicitis, reporting the type of appendicitis or a subgroup analysis is very important to interpret complication rates. Unfortunately studies often do not report the definitions used to classify simple or complex appendicitis, or the rates of simple or complex appendicitis are missing altogether. In this study, we chose to adhere to the most widely accepted definition for simple and complex appendicitis and to review all operative and histopathological reports to classify the type of appendicitis [2]. To improve future comparability of studies universal reporting of appendicitis severity and its complications is of vital importance.

## Conclusion

Results of this study show a high rate of complications compared with current literature, reflecting a significant burden of disease, even in children with simple appendicitis. These results challenge the present notion that an appendectomy is a routine procedure with only few complications. However, most importantly, universal reporting of appendicitis type and its complications is needed in order to drastically improve the comparability of future studies, which is a key element to further improve the care for children suffering from appendicitis.

## Data Availability

All available data and statistical analyses can be made available to other researchers pending approval of a research proposal by the APAC study team. Researchers can contact the corresponding author to make such a request.
